# Heme Oxygenase-1: Its Therapeutic Roles in Inflammatory Diseases

**DOI:** 10.4110/in.2009.9.1.12

**Published:** 2009-02-28

**Authors:** Hyun-Ock Pae, Hun-Taeg Chung

**Affiliations:** Department of Microbiology and Immunology, Wonkwang University School of Medicine, Iksan, Korea.

**Keywords:** heme oxygenase-1, carbon monoxide, bilirubin/biliverdin, inflammation, nuclear factor E2-related factor-2, mitogen-activated protein kinase

## Abstract

Heme oxygenase (HO)-1 is an inducible enzyme that catalyzes the first and rate-limiting step in the oxidative degradation of free heme into ferrous iron, carbon monoxide (CO), and biliverdin (BV), the latter being subsequently converted into bilirubin (BR). HO-1, once expressed during inflammation, forms high concentrations of its enzymatic by-products that can influence various biological events, and this expression is proven to be associated with the resolution of inflammation. The degradation of heme by HO-1 itself, the signaling actions of CO, the antioxidant properties of BV/BR, and the sequestration of ferrous iron by ferritin all concertedly contribute to the anti-inflammatory effects of HO-1. This review focuses on the anti-inflammatory mechanisms of HO-1 actions and its roles in inflammatory diseases.

## INTRODUCTION

Heme is inherently dangerous when, in excessive amounts, released from intracellular heme-containing proteins (1). The released heme, or free heme, may cause oxidative and inflammatory injury associated with the pathology of diverse conditions (2). Thus, it is of most importance to remove excess of free heme at sites of injury. The microsomal enzyme heme oxygenase (HO) catalyzes the first and rate-limiting step in the oxidative degradation of free heme to produce carbon monoxide (CO), ferrous iron (Fe^2+^), and biliverdin (BV) (3). BV formed in this reaction is subsequently converted into bilirubin (BR) by a BV reductase, and the ferrous iron is rapidly sequestered by ferritin and recycled for heme synthesis (4). To date, two genetically distinct isozymes of HO have been characterized: an inducible form, heme oxygenase-1 (HO-1), and a constitutively expressed form, heme oxygenase-2 (HO-2) (5). Although both HO-1 and HO-2 catalyze the identical biochemical reaction, there are some fundamental differences between the two in genetic origin, primary structure, and molecular weight (4). HO-1, once expressed under various pathological conditions, has an ability to metabolize high amounts of free heme to produce high concentrations of its enzymatic by-products that, as such a consequence, can influence various biological events, and has recently been the focus of considerable medical interest (6). HO-1 can be expressed not only by its substrate, free heme, but also by a wide variety of pro-inflammatory stimuli, suggesting that HO-1, besides its fundamental role in heme degradation, plays other important roles in resolution of inflammation (7). In this regard, this review has focused on the anti-inflammatory mechanisms of HO-1 actions and its roles in inflammatory diseases. This information is important for the development of potential drugs that may alleviate the numerous inflammatory diseases through activation of HO-1 expression.

## REGULATION OF HO-1 EXPRESSION

There may be the potential interplay between intracellular and nuclear signaling events that lead to transcriptional activation of the *ho-1* gene by various stimuli. Various transcription factors, including the nuclear factor E2-related factor 2 (Nrf2), interact with their cognate DNA binding domains in the HO-1 promoter to up-regulate *ho-1* gene transcription, and a number of intracellular signaling molecules, including the mitogen-activated protein kinase (MAPK), involve the activation of these transcription factors (8,9). The MAPK family comprises three primary signaling cascades: the extracellular signal regulated kinase (ERK), the c-Jun NH_2_-terminal kinase (JNK), and the p38 MAPK. Depending on the specific stimulus and the cell type involved, one or more of MAPK pathways may involve HO-1 expression (10). The involvement of Nrf2 in HO-1 expression has been highlighted by the finding that HO-1 is less inducible in Nrf2-deficient mice (11). It should be noted that Nrf2 activation in response to the specific stimulus generally requires MAPK activation. For examples, Kim and co-workers (12) demonstrated that 15-Deoxy-Δ^12,14^-prostaglandin J_2_ induced HO-1 expression through activation of ERK and other kinase pathways that leads to Nrf2 activation. The antioxidant quercetin protected human hepatocytes from ethanol-induced oxidative stress via Nrf2- dependent HO-1 expression, and p38 MAPK and ERK mediated quercetin-induced Nrf2 activation (13).

## REACTION PRODUCTS OF HO-1 AND THEIR ANTI-INFLAMMATORY ROLES

**Figure 1:**
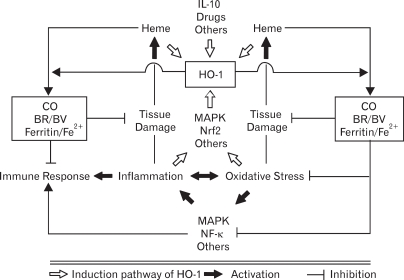
Induction of HO-1 and subsequent production of heme degradation products exert potent anti-oxidative, anti-inflammatory and anti-apoptotic functions for the tissue homeostasis. HO-1 can be expressed by a number of stimuli mainly via MAPK-dependent Nrf2 activation. These inducers of HO-1 include free heme, inflammatory mediators, oxidative stress, IL-10, and some inflammatory drugs. HO-1, once expressed under pathological conditions, can degrade free heme into BV, CO, and Fe^2+^. BV is converted into BR by BV reductase. The iron is rapidly sequestered by ferritin. Heme degradation products have been shown to modulate inflammatory response, perhaps by reducing oxidative stress, blocking MAPK pathways, and suppressing NF-κB activity.

HO-1 expression is up-regulated in response to various forms of inflammatory stimuli, and this is associated with reduced inflammation (14). However, the mechanism(s) of anti-inflammatory actions of HO-1 has not been completely elucidated. It is most likely that the anti-inflammatory effects afforded by HO-1 may be attributed not only to its own action but also to other actions of three by-products of HO-1 activity (6). On other words, the degradation of the pro-oxidant heme by HO-1 itself, the signaling actions of CO, the antioxidant properties of BV/BR, and the sequestration of free iron by ferritin could all concertedly contribute to the anti-inflammatory effects observed with HO-1 (Fig. 1). The followings briefly describe the mechanisms that may explain the anti-inflammatory actions of HO-1.

### CO

The gaseous molecule CO seems to be responsible for most of the anti-inflammatory actions of HO-1 (15). In macrophages, CO inhibited the production of pro-inflammatory cytokines, such as tumor necrosis factor-α (TNF-α), interleukin-1β (IL-1β), and macrophage inflammatory protein-1, through modulation of p38 MAPK activation (16). In human T cells, CO suppressed IL-2 secretion and clonal expansion via inhibition of ERK pathway (17). CO also suppressed the expression of pro-inflammatory enzymes, such as inducible nitric oxide synthase and cyclo-oxygenase-2, in macrophages by virtue of its ability to regulate the C/EBP and nuclear factor-κB (NF-κB) activation (18). In human colonic epithelial cells, the inhibitory effects of CO on iNOS expression and IL-6 secretion were dependent on the modulation of NF-κB, activator protein-1 (AP-1), C/EBP activation, and MAPK pathway (19).

### BV/BR

The natural antioxidant BV/BR appears to substitute for some anti-inflammatory effects of HO-1 (20). The anti-inflammatory effects of BV on organ transplantation are manifested by decreased leukocyte infiltration, less T cell proliferation, and extended survival of allogeneic heart transplants (21). In the rat model of endotoxin-induced shock, BV reduced serum levels of the pro-inflammatory cytokines, but enhanced the production of the anti-inflammatory cytokines (22). BR inhibited endothelial cell activation by suppressing E-selectin and vascular cell adhesion molecule-1 expression (23). A possible mechanism by which these effects of BV/BR occur may involve the inactivation of NF-κB, a transcription factor strictly required for the transcription of the pro-inflammatory genes (22,23).

### Ferritin expression

The expression of ferritin is markedly enhanced in conjunction with HO-1 expression (24). Although whether the ferritin could substitute for anti-inflammatory actions of HO-1 is not completely elucidated, it has been proven to be a potent antioxidant enzyme (25). Ferrous iron, an extremely pro-oxidative molecule that may cause inflammation, is released during the breakdown of free heme by HO-1 (24). In that case, the ferritin effectively sequesters the ferrous iron and, hence, limits its pro-oxidant/pro-inflammatory capacity (24). Based on the data presented by Schaer and co-workers (26), the induction of HO-1 was directly involved in the down-regulation of inflammation, and this effect was due to ferritin synthesis as a result of HO-1 induction.

## LESSONS FROM HO-1-DEFICIENT MICE

HO-1 plays important roles at least in the resolution of inflammation, and this concept is represented by the two genetic findings: 1) the mice with HO-1 deficiency have a distinct phenotype of an increased inflammatory state (27,28) and 2) an inflammatory syndrome that developed in a HO-1-deficient human patient was one of the reasons of death (29,30). Kapturczak and co-workers (27) examined the differences in immune phenotype between HO-1 knockout (HO-1^-/-^) and wild-type (HO-1^+/+^) mice. The first finding is that a deficiency of HO-1 may predispose to generally exaggerated inflammatory responses, suggesting that its activity is necessary for timely resolution of early inflammation. The other is that HO-1^-/-^ splenocytes secreted disproportionately higher levels of pro-inflammatory cytokines as compared to those from HO-1^+/+^ mice, suggesting that HO-1 activity is also important in more downstream stages of the immune response. These two findings raise an important question as to how HO-1 can modulate the immune response.

CD4^+^CD25^+^ regulatory T (Treg) cells maintain immunological self-tolerance and limit the deleterious effects associated with inflammatory reactions (31). Interestingly, there are the similarities in the anti-inflammatory functions attributed to Treg cells and to HO-1 activity, raising a possibility that HO-1 would be a key mediator of activities of Treg cells. Two studies have tested this hypothesis through the use of HO-1^-/-^ mice. Zelenay and co-workers (31) have demonstrated that HO-1 is not essential for mouse Treg development, maintenance and function. On the contrary, George and co-workers (32) have demonstrated that a lack of HO-1 in antigen-presenting cells (APCs) significantly impairs the suppressive function of Treg cells under conditions of APC excess. The later would provide an explanation for the immunoregulatory defects observed in HO-1^-/-^ mice.

## ROLES OF HO-1 IN INFLAMMATORY DISEASES

In addition to valuable lessons from HO-1-deficient mice, the aforementioned properties of HO-1 and its by-products provoked researchers' interest in investigating the impact of HO-1 on the development of inflammatory diseases in animal models. Indeed, the cytoprotective effects of HO-1 have been well confirmed in a number of experimental models, including sepsis, transplantation, autoimmunity, and allergy.

### Sepsis

Sepsis is a systemic inflammation against severe infection, which contributes to the cascade of events that ends not only in shock but also in multiple organ dysfunction and death. Despite improved therapy and better understanding of the mechanisms underlying its pathogenesis, sepsis remains to be a leading cause of morbidity and mortality in the intensive care unit.

Symptoms of Gram-negative bacterial sepsis can be reproduced experimentally by administration of animals with lipopolysaccharide (LPS), a major component of the outer membrane of Gram-negative bacteria. In such a sepsis model, macrophages are one of the cells that are most sensitive to LPS stimulation. Once activated in response to LPS, macrophages release endogenous mediators and defense molecules, including pro-inflammatory cytokines such as TNF-α. The pro-inflammatory cytokines could protect the host against bacterial infection, but an exaggerated stimulation of the immune system with a marked release of cytokines can lead to hypotension, collapse of circulatory system, multiple organ dysfunction, and death. The extent of cellular damage in the sepsis may be determined not only by the pro-inflammatory cytokines and enzymes but also by the anti-inflammatory cytokines and enzymes. Among the anti-inflammatory enzymes, HO-1 has been shown to mediate protective effects in LPS-induced sepsis model: there is accumulating evidence emphasizing the importance of HO-1 in the development of sepsis (33-41). It has been suggested that administration of mice with LPS was associated with a marked increase HO-1 gene expression in a site specific organ manner (42). Interestingly, HO-1 expression in monocytes from patients with severe sepsis significantly increased, as compared with that in monocytes from healthy volunteers (43). The possible roles of HO-1 in LPS-induced sepsis have been well characterized using HO-1-deficient mice. HO-1-deficient mice develop increased end-organ damage and have increased mortality after LPS administration (41). In contrast, administration of CO to HO-1-deficient animals attenuates LPS-induced inflammation and end-organ injury (44). These studies support the beneficial effects of HO-1 and its by-products such as CO during sepsis.

### Graft rejection

Organ transplantation has become an ultimate therapeutic option for irreversible organ failure. Early graft survival has significantly improved; however, the long-term outcome remains unsatisfactory. Long-term graft function and graft survival are affected by both non-immunologic and immunologic factors. Ischemia reperfusion (IR) injury represents the major non-immunologic factor implicated in the pathogenesis of graft dysfunction and activation of alloreactive T cells, mainly due to failure of transplantation tolerance, is served as the major immunologic factor.

There is accumulating evidence to support the notion that HO-1 expression in a graft and in the recipient can prevent graft rejection and promote immune tolerance (45-57). Under a given immunosuppressive regimen, the survival of a mouse heart transplanted into a rat was related to high levels of HO-1 expression in the graft vasculature (58). Hearts from HO-1-deficient mice failed to survive when transplanted under the same immunosuppressive regimen (51), suggesting that HO-1 expression in transplants from wild-type mice contributes in a critical manner to sustain their survival. This notion has now been expanded by showing that HO-1 expression can inhibit different processes involved in IR injury as well as in the acute and chronic rejection of transplanted organs (59). IR injury triggers a pro-inflammatory response in the graft, which in turn augments graft immunogenicity and as such results causes graft dysfunction. Endogenous HO-1 expression inhibited IR injury during organ transplantation (60), and this is the first explanation regarding how HO-1 can promote graft survival. The protective effects of HO-1 may be mediated via several pathways: removal of free heme generated during IR injury, antioxidant properties of BV/BR, and vasoregulatory effects as well as anti-inflammatory, anti-apoptotic, and antiproliferative properties of CO. The second explanation is that HO-1 was also involved in transplantation tolerance (61). The tolerogenic effect of HO-1 was found dependent on the activation of CD4^+^CD25^+^ Treg cells (51).

### Autoimmune disease

Autoimmunity occurs when the immune system recognizes and attacks host tissue. In addition to genetic factors, environmental triggers are thought to play a major role in the development of autoimmune diseases. Autoimmune diseases fall into two general types: those that damage many organs (systemic autoimmune diseases) and those where only a single organ or tissue is directly damaged by the autoimmune process (localized). Examples of autoimmune diseases include type I diabetes, multiple sclerosis, rheumatoid arthritis (RA), and lupus.

HO-1 is considered as an endogenous factor responsible for the resolution of inflammation and might thus be a novel target for the modulation of the inflammatory autoimmune response. Recently, several investigators have presented evidence to support an immunosuppressive function of HO-1 during the course of autoimmune diseases (62-69). Hu and co-workers (64) evaluated the effect of HO-1 on autoimmune diabetes. Using NOD mice which spontaneously develop type I diabetes, they showed that intravenous HO-1 transduction reduced destructive insulitis and the incidence of overt diabetes by down-regulating the phenotypic maturity of dendritic cells and Th1 effector function. A similar protective effect against diabetes was also observed in NOD mice subjected to CO (70). Chora and co-workers (66) reported that HO-1 expression dictated the pathologic outcome of experimental autoimmune encephalomyelitis (EAE), a model of multiple sclerosis, and pharmacological induction of HO-1 suppressed the pathologic outcome of autoimmune neuro-inflammation associated with the development of EAE, presumably by inhibiting the expression of MHC class II on APCs and the reactivation of pathogenic CD4^+^ T cells within the central nerve system. Kobayashi and co-workers (67) examined the expression and pathogenetic roles of HO-1 in RA, and found that HO-1 expressed in RA synovial tissues protected against the onset of RA.

### Allergic disease

Allergic reactions differ from protective immune reactions (immunity) only in that they are exaggerated or inappropriate and damaging to the host (hypersensitivity). The cellular and molecular mechanisms of the two types of reaction are virtually identical. The sequence of events involved in the development of allergic reaction can be divided into three phases: the sensitization phase, during which immunoglobulin E (IgE) is produced in response to allergenic stimulus and binds to specific receptors on mast cells and basophils; the activation phase, during which re-exposure to allergen triggers the mast cells and basophils to release of the contents of their granules; and the effector phase, during which an anaphylactic response occurs as a result of release of pharmacologically active agents. After subsidence of acute reaction, localized inflammation is elicited by the mediators released during the course of immediate reaction as late-phase reaction.

HO-1 expression is up-regulated in allergic triad (asthma, allergic rhinitis, and atopic dermatitis), and HO-1 has been proven to be anti-allergic (71). Almolki and co-workers (72) reported the anti-allergic role of HO-1 in allergic airway inflammation. The guinea pigs were sensitized with ovalbumin (OVA) to develop characteristic features of asthma, and treated with the HO-1 inducer hemin during sensitization or after developing impaired airways features. Oxidative stress in OVA-challenged animals and the number of neutrophils, eosinophils, and lymphocytes in their airways were markedly reduced when HO-1 expression was induced by hemin, suggesting a protective role of HO-1 against airway inflammation. Kirino and co-worker (73) have demonstrated that serum HO-1 levels are increased in patients with atopic dermatitis (AD) and that the levels correlate with the severity of clinical manifestations and conventional disease markers. In an AD mouse model, HO-1 was abundantly expressed in resident macrophages and DCs in the AD-like skin lesions. Interestingly, pharmacologic induction of HO-1 suppressed the development of these AD-like skin lesions, suggesting a protective role of HO-1 against skin inflammation. At present, the exact mechanisms by which HO-1 could exert anti-allergic effects have not been completely elucidated. In addition to the antioxidant/anti-inflammatory properties of HO-1 and its by-products, blockage of mast cell activation (74), inhibition of IgE production (75), and modulation of immunological functions of T cells and DCs (76) might mediate anti-allergic effects of HO-1.

## CONCLUSION

During the last decade, the beneficial roles that HO-1 could play in a number of disease states have been elucidated, and the protective/anti-inflammatory role of HO-1 has been highlighted. In the present review, we have briefly summarized the current data confirming the beneficial effects of HO-1 on inflammatory diseases. It is now commonly accepted that HO-1 plays a crucial role in the resolution of inflammation (71,77). For examples, mice lacking HO-1 were more susceptible to inflammatory injury (27,28), whereas the use of pharmacological agents and genetic probes for up-regulating HO-1 expression rendered experimental animals less susceptible to inflammation (33-43). Although the exact mechanism(s) by which HO-1 can exert anti-inflammatory effects has not yet been elucidated, its metabolic products have been shown to mimic anti-inflammatory actions of HO-1. The use of CO and BV/BR as therapeutic agents has been successful in some inflammatory models. Based on the current researches focusing on the anti-inflammatory roles of HO-1 or its metabolic products, we suggest that the strategies to target HO-1 or its metabolic products may offer promising therapeutic approaches for the effective management of a number of inflammatory diseases.
